# Post-harvest Industrial Processes of Almond (*Prunus dulcis* L. Mill) in Sicily Influence the Nutraceutical Properties of By-Products at Harvest and During Storage

**DOI:** 10.3389/fnut.2021.659378

**Published:** 2021-06-02

**Authors:** Chiara Caltagirone, Cristiana Peano, Francesco Sottile

**Affiliations:** ^1^Department of Architecture, University of Palermo, Palermo, Italy; ^2^Interdepartmental Research Center in Bio-Based Reutilization of Waste from Agri-Food Matrices (RIVIVE), University of Palermo, Palermo, Italy; ^3^Department of Agricultural, Forestry and Food Sciences, University of Turin, Turin, Italy

**Keywords:** almond, skin, phenols, antioxidant, storage

## Abstract

Almond cultivation in Sicily is experiencing a phase of great interest which is mainly concentrated in the development of specialized orchards, with irrigation and by adopting cultivars with high qualitative and quantitative performances. These are mostly Mediterranean genotypes with high fat content and hard or semi-hard shell, extremely different from the varieties of Californian diffusion. The development of the sector comprises the primary production of almonds but also a series of secondary products which often represent a burden for the company. From these considerations several researches have been developed with the aim of giving a value to these by-products through circular economy paths. One of the by-products widely produced, besides the shell, is the skin which covers the seed and is produced during the peeling phase. It is well-known that tegument is an important component of almond because it contains important bioactive substances (phenols and aromas) which are usually dispersed during peeling. This paper examined three different Italian cultivars widely spread in Sicily, two of Apulian origin, (Genco and Tuono), and one locally cultivated variety (Vinci a tutti). These three cultivars occupy an increasingly large area and are very popular with consumers and industry. The production of secondary products, therefore, evidences significant quantities and the possibility to give them an added value becomes a need for the sector. Therefore, the content of phenols and proanthocyanins in the skin at harvest and during storage was analyzed, adopting two different separation methods, with and without water. During the analysis it was possible to observe the different behavior of the three cultivars due to genetic and industrial factors. Skin separated without using hot water showed a higher total phenolic presence with average increases of about 20%, and with even higher increases, between 28 and 32%, for proanthocyanins. Vinci a tutti evidenced the highest total phenolic content after 8 months of storing while roasting has revealed to be a more effective skin separation approach.

## Introduction

Although almond cultivation, in the Mediterranean basin, has been practiced for several centuries, the increasing consumption, combined with the proven nutraceutical properties, have attracted the attention of many researchers not only from the agronomic sector, but also from the pharmaceutical and biomedical ones (1). In this context, a steady growth of preference for almonds from Italy has been observed (2). There are many and different causes that have led to this revaluation; among them certainly the recent contribution of the research in terms of agronomic advances, the reduction of the production area in the USA and the evolution of international markets and consumption on the domestic market (3). In Italy, a country with a strong vocation for almond cultivation, a large part of the total production can be attributed to Sicily.

For about a decade, there has been a coexistence in Sicily between two types of almond cultivation, (i) a modern one, certainly more competitive at the international standards, which combines modern knowledge in the field of fruit cultivation by adapting it to local varieties, and (ii) a traditional almond cultivation, often linked to a more historical and landscaping function, and based on old poorly competitive varieties but with a high scientific interest for genetic improvement (4–6).

It is also evident that the new approach to innovative almond orchards is designed considering all issues related to the protection and preservation of the environment, mostly in terms of total sustainability, in full compliance with the 2030 Agenda and the Sustainability Development Goals (7). A specialized agricultural model, in fact, is usually more demanding in terms of production inputs (pesticides, fertilizers, etc.) mostly due to a higher number of trees per hectare and a higher yields.

In recent years there has been a positive surge in nuts marketing, especially in highly developed countries where frequent dietary dysfunctions promoted the urgent need to change the current lifestyle (8). This upsurge is precisely because of profile of bioactive substances these offer that have led to reevaluation of nuts consumption worldwide. Among the world's nuts productions, a major role is played by almonds, followed then by Brazil nuts, cashews, hazelnuts, macadamias, pecans, pine nuts, pistachios and walnuts (9).

Biochemical composition of almond is influenced by exogenous and endogenous factors. In fact, if a fundamental role is played by the cultivar, it is not possible to ignore the importance of temperature, year of production, location and cultivation techniques adopted during cultivation. For example, irrigation positively influence the quantity of almonds, while it has a limited effect on the quantity of micro and macronutrients contained in them (10). Most of these nutraceuticals are found in both the seed and its tegument (11, 12).

When the almond is sent for industrial processing, it is, first of all, dehulled and then unshelled in order to obtain only the edible part. Almonds can therefore be consumed as they are with their tegument or they can undergo further processing and transformations. It is therefore evident that the percentage of by-products derived from almonds processing is quite high and this determines the necessity of their disposal and alternative use. Currently they are mainly used as feed for livestock or for the production of energy through gasification, obtaining fuel from almonds shells.

The use of almonds is quite traditional and diffuse. In Italy, as well as in many Mediterranean Countries, almonds are consumed as snacks but they also play a very important role in traditional confectionery (13). This dry fruit, in fact, plays an important role for its nutritional properties and its typical presence in the warm environments of the Mediterranean has determined its diffusion attributing a relevant role in nutritional paths related to the Mediterranean diet (14). In many traditional recipes related to rural culture, almonds are mainly used peeled, therefore without the skin (15, 16). Only more recently there are some interesting scientific evaluations on the importance of by-products that are also finding application in local and industrial processing (17). In the previous years, some producers of almond based beverages are making interesting trials using unpeeled almonds mainly for the preservation of aromatic substances.

As previously mentioned, the almond skin is also rich in nutraceutical substances as shown by many studies (11, 18–25) that classified the teguments by their content of hydroxybenzoic acids and aldehydes, hydroxycinnamic acids, flavan-3-ols, flavanols, dihy-droflavonols, and flavanones.

All of these compounds have numerous beneficial properties for human health. Among them the antioxidant, anti-inflammatory, anti-allergic, antiviral, anticarcinogenic, and anti-cholesterolemic properties are the most relevant (26–30). Potential of almond by-products and their nutraceuticals have been previously studied by using different methodologies (31, 32).

Based on these evidences, the almonds skin could be used to obtain compounds with good antioxidant properties, which can be used as additives to control oxidative processes in the food industry or as functional ingredients in food supplements. It seems therefore interesting to understand the industrial approach for the skin separation. In the same way, this study is also addressed to evidence the effect of almond storage by adopting different methodologies.

## Materials and Methods

The trials were carried out considering the three most widespread Italian cultivars in the territory of Mazzarino (Caltanissetta Province, Sicily, Italy), “Tuono,” “Genco” and “Vinci a tutti.” The first two are of Apulian origin, but now also widely used in Sicilian almond cultivation. The third one is of Sicilian origin, traditionally spread in the area of research.

Almond samples were obtained from a randomized orchard from almond trees present in the territory. Innovative mature orchards have been chosen, with plants grafted on seedlings, equipped with micro-flow irrigation system. The orchards belong to the same company and, therefore, they are managed through similar management techniques related to soil, irrigation, harvesting and post-harvest processing. Harvesting is mechanized, carried out with the aid of a self-propelled shaker with inverted umbrella conveyor.

Ten plants were selected within the plot for each cultivar; at harvest, the in-shell yield per tree was weighed and, after sun drying, the shelling rate and the fraction attributable to the skin were measured.

For the experiments during storing, the sampling protocol was applied at different times of the year, considering the shelf life of the nuts. Therefore, the sampling phases were:

- harvest;
- 120 days after harvest (dah);
- 240 dah.

Nut storage was accomplished in two different ways:

- in shell, in 1,000 kg big bags, at a controlled temperature of 20°C ±3;
- shelled, in 25 kg vacuum packs, at a controlled temperature of 6°C ±1.

In the latter case, vacuum seed packages were made immediately after shelling at harvest.

Almonds from the three cultivars were processed by two different treatments for skin separation:

- blanching at 95°C for 3 min followed by peeling;
- roasting at 145°C for 14 min in a ventilated oven with continuous cycle and subsequent skin separation.

**Figure 1:**
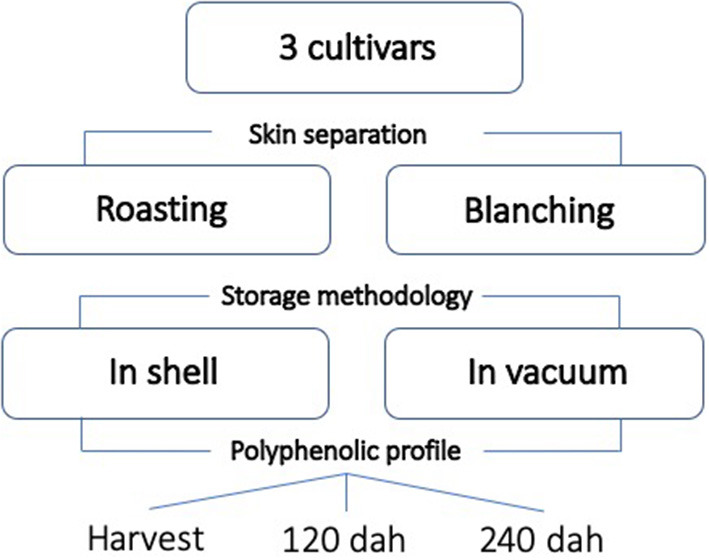
Experimental framework reporting all the treatments and the variables applied (dah: days after harvest).

The complete experimental framework is reported in Figure 1.

In the case of blanching, after removal the teguments are immediately dried in a hot-air oven at 60°C until they reach a constant moisture content. The overall moisture content achieved by the skin samples ranged from 4 to 5%.

Skin samples from each treatment were then ground in particles ≤ 0.5 mm in a mill (IKA Multidrive-control) and the antioxidant fraction was extracted with 10 ml of methanol/HCl from 50 mg of ground teguments. The mixture was then centrifuged and filtered (0.45 μm) to determine the total phenolic content according to the methodology of Singleton and Rossie (33).

The antioxidant fraction was determined by the ORAC (Oxygen Radical Absorption Capacity) method which allows to measure how the food antioxidants work in synergy determining a real antioxidant potential. The ORAC value therefore measures how effective these antioxidants are in neutralizing free radicals, and thus combat oxidative stress that is the cause of aging and other diseases. The ORAC value was obtained using fluorescein as a fluorescent probe by using the Zen-Bio (Research Triangle Park, NC, USA) ORAC kit. The reaction took place at a temperature of 37°C with 75 mM phosphate buffer at pH 7.4. The final mixture (200 μL) contained: fluorescein (70 nM), 2,2′-azobis (2-methylpropionamidine), dihydrochloride (12 mM), and antioxidant [Trolox (1–8 μM) or sample (at different concentrations)]. Fluorescence was recorded every minute for 98 min after an automatically removing of the plate. All fluorescence measurement equipment was controlled by Fluostar Galaxy software and equated to the empty curve (without antioxidant). The final ORAC values expressed in mM of TE equivalent per gram of the sample taken as an example.

The data were analyzed using two-ways ANOVA test. Means separation was performed by using Tukey test and were considered significant at *p* < 0.01. Analyses were carried out using SPSS v. 22.0 (IBM Corporation, New York, SA). Interactions were found not significant.

## Results and Discussion

Field and primary processing observations of almonds showed differences in terms of production and yield of the different cultivars: “Genco” and “Tuono” were the most productive varieties, with average yields per tree of 15 and 14 kg, respectively, compared to “Vinci a tutti,” 12 kg; thanks to a lower shell hardness they also showed a higher yield in shelled almonds. The shell hardness is always measured as the ratio between the ratio between seed weight and whole fruit weight (4). “Tuono” and “Genco” had similar kernel weight (1.4 g on average) while “Vinci a Tutti” a bit heavier (1.6 g on average). The difference on the percentage of skin appeared statistically significant (Table 1).

**Table 1:** Yield, shelling rate, and skin percentage in three Italian almond cultivars.

**Cultivar**	**Yield in shell**	**Shelling rate**	**Skin**
	**kg/tree**	**%**	**%**
Genco	15, 1.7 *n.s*	34.7 ± 0.4 b	5.2 ± 0.01 a
Tuono	14, 1.1	37.2 ± 0.5 a	5.1 ± 0.01 b
Vinci a tutti	12, 1.7	31.2 ± 0.7 c	4.1 ± 0.01 c

*Different letters within column evidence differences statistically significant per P < 0.01; n.s., not significant*.

The polyphenolic profile of almonds mainly comprises of proanthocyanidins followed by hydrolysable tannins, flavonoids, and other minor compounds. Procyanidin B2 and procyanidin B1 are the predominant with a wider range in whole almonds. Procyanidin B2 is the one with the highest range when considering separated skin (34).

As regards total polyphenols and proanthocyanins, in this study there is a constant higher content in the cultivar “Vinci a tutti” compared to “Genco” and “Tuono” with differences that frequently oscillate between 10 and 15%, during all the testing phases.

Cultivar aspects being equal, a fundamental role in the concentration of total polyphenols and proanthocyanins is played by the industrial treatment for skin separation. In fact, skin separated without using hot water showed a higher total phenolic presence with average increases of about 20%, and with even higher increases, between 28 and 32%, for proanthocyanins. The concentration of phenolic compounds of the seed skin alone is in consonance with previous findings on whole almonds (35–37).

These values are comparable and even higher than those found in the literature after applying the same extraction methodology to the skins of wine grapes, whose by-products are commonly used in food industry as ingredients for the production of food supplements of antioxidant nature (38).

The high phenolic content in the skin separated from roasted almonds is probably due to the fact that during blanching in water many of the phenolic compounds are solubilized, resulting in a loss of total content. First non-experimental evaluations have also highlighted a very high fermentative power of blanching water probably due to a relevant content of glycosylated favonoids and proanthocyanins (39). Moreover, hot processing would increase the total phenolic content of the skin which are therefore more concentrated after roasting. The phenolic content after roasting shows in literature conflicting data even in case of a similar process. Some authors, indeed, reported an initial decrease in phenolic content immediately after roasting with subsequent recovery during storage (40). Other authors reported a consistent increase affected by separation methods (41) and this behavior can be probably interpreted as the increase of the extractable phenolic compounds post roasting (42).

The results show, therefore, that for all cultivars, although there are differences between them, the most suitable type of industrial processing of almonds to obtain extracts from almond skin with the highest antioxidant capacity at harvest is roasting (Table 2).

**Table 2:** Total polyphenols in the seed skin of three Italian almond cultivars at harvest.

**Cultivar**	**Post-harvest treatment**	**Polyphenols**	**Proanthocyanins**	**ORAC**
		**mg/g**	**mg/g**	**mmol Trolox/g**
Genco	Blanching	25.5, 1.6 ab	30.4, 1.9 a	0.499, 0.040 a
Tuono	Blanching	24.7, 1.2 b	29.1, 2.1 a	0.477, 0.039 a
Vinci a tutti	Blanching	26.4, 1.1a	32.5, 2.4 a	0.510, 0.037 a
Genco	Roasting	32.1, 2.6 ab	43.3, 2.4 ab	0.844, 0.032 a
Tuono	Roasting	31.4, 2.4 b	40.6, 2.7 b	0.821, 0.031 a
Vinci a tutti	Roasting	33.6, 2.1a	44.7, 2.8 a	0.860, 0.033 a

*Different letters between cultivars within the same storage duration and post-harvest treatment evidence differences statistically significant per P < 0.01 (Tukey's test)*.

The ORAC follows, as it is to be expected, a behavior comparable to that of total polyphenols and proanthocyanins; it is therefore found a consistently higher value in the skin of roasted almonds than in those peeled after water treatment (Tables 2, **5**).

This shows in some sense that the loss of total polyphenols is consistently proportional to the antioxidant power of the substrate obtained.

As regards the post-harvest storage of almonds, another interesting finding was recorded by comparing the content of phenolic compounds of samples preserved in shell and those preserved in vacuum, concluding that the preservation in shell was more effective in terms of lower loss of these properties over time.

In fact, it is well-known that in-shell almonds, if well-preserved, remain edible after storage up to more than 1 year (43) and this would seem to be influenced also by their vitamin E content, higher concentration means higher shelf-life (44, 45). Other studies have identified the 18th month of storage as the time when degradative processes begin, precisely because after 18 months almonds lose a significant percentage of vitamin E, about 90% (46, 47). Peeling and exposure to high temperatures, light, and oxygen decrease the shelf life of almonds and promote lipid oxidation and the formation of off-flavors with widespread fermentation (rancidity) (48, 49).

Contrary to our hypothesis that storage significantly reduces the phenolic fraction of the almond, the data showed quantitatively anomalous results. The measurements after 4 and after 8 months from harvesting with two different storing methods, in all treatments and for all cultivars there was a decrease, although not extremely marked and not always statistically significant, in the content of total polyphenols, proanthocyanins and ORAC fraction (Tables 2–5). “Vinci a tutti,” which started with a higher phenolic content, maintained it up to 8 months after harvest as well as for the values of proanthocyanins (retention percentage 96.5 and 95%, respectively). Almonds stored in shell showed a less marked total phenolic and qualitative decrease, while for ORAC the reduction was extremely relevant. (“Vinci a tutti,” in this case, shows a collapse between 120 and 240 days both in the case of in-shell storage and in the case of vacuum storage of almonds and, similarly, both in the case of separation of the skin with roasting and peeling, although the starting values were substantially different from those of other cultivars. This behavior testifies in some way to a lesser attitude of the local cultivars for long-term preservation which, both in shell and in vacuum, highlights a series of critical points which are revealed only through in-depth analysis (Tables 2–5).

**Table 3:** Polyphenols in the seed skin of three Italian almond cultivars at 120 and 240 dah during in-shell and vacuum storage.

**Cultivar**	**Post-harvest storage**	**Post-harvest treatment**	**120 dah**	**240 dah**
			**mg/g**	**mg/g**
Genco	In-shell	Blanching	25.1, 1.5 ab	24.2, 1.4 a
Tuono	In-shell	Blanching	24.0, 1.1 b	23.1, 1.1 a
Vinci a tutti	In-shell	Blanching	26.1, 1.0 a	25.5, 1.3 a
Genco	In-shell	Roasting	31.1, 2.4 a	30.7, 2.1 a
Tuono	In-shell	Roasting	30.9, 2.1 a	29.9, 2.3 a
Vinci a tutti	In-shell	Roasting	32.9, 1.8 a	32.0, 2.2 a
Genco	In-vacuum	Blanching	24.8, 1.5 a	24.1, 1.5 a
Tuono	In-vacuum	Blanching	24.0, 1.1 a	23.3, 1.2 a
Vinci a tutti	In-vacuum	Blanching	25.9, 1.0 a	25.4, 1.2 a
Genco	In-vacuum	Roasting	31.0, 2.5 ab	30.5, 2.1 a
Tuono	In-vacuum	Roasting	30.5, 2.1 b	29.9, 2.4 a
Vinci a tutti	In-vacuum	Roasting	32.7, 1.9 a	32.1, 2.2 a

*dah, days after harvest—Different letters between cultivars within the same storage duration and post-harvest treatment evidence differences statistically significant per P < 0.01 (Tukey's test)*.

**Table 4:** Proanthocyanins in the seed skin of three Italian almond cultivars at harvest and during in-shell and vacuum storage.

**Cultivar**	**Post-harvest storage**	**Post-harvest treatment**	**120 dah**	**240 dah**
			**mg/g**	**mg/g**	
Genco	In-shell	Blanching	29.7, 1.6 ab	27.8, 1.4 ab
Tuono	In-shell	Blanching	27.9, 2.0 b	25.9, 2.2 b
Vinci a tutti	In-shell	Blanching	32.0, 2.3a	31.1, 2.1 a
Genco	In-shell	Roasting	43.1, 2.2a	42.2, 2.3 a
Tuono	In-shell	Roasting	39.7, 2.4 b	38.1, 2.2 b
Vinci a tutti	In-shell	Roasting	44.1, 2.6a	43.6, 2.7 a
Genco	In-vacuum	Blanching	28.9, 1.7a	27.7, 1.5 ab
Tuono	In-vacuum	Blanching	27.4, 2.1a	25.7, 2.2 b
Vinci a tutti	In-vacuum	Blanching	31.8, 2.4a	31.3, 2.1 a
Genco	In-vacuum	Roasting	42.8, 2.5a	42.0, 2.2 a
Tuono	In-vacuum	Roasting	39.5, 2.5 b	37.6, 2.1 b
Vinci a tutti	In-vacuum	Roasting	44.3, 2.6a	43.3, 2.8 a

*dah, days after harvest—Different letters between cultivars within the same storage duration and post-harvest treatment evidence differences statistically significant per P < 0.01 (Tukey's test)*.

**Table 5:** ORAC in the seed skin of three Italian almond cultivars at harvest and during in-shell and vacuum storage.

**Cultivar**	**Post-harvest storage**	**Post-harvest treatment**	**120 dah**	**240 dah**
			**mmol Trolox/g**
Genco	In-shell	Blanching	0.487, 0.035a	0.475, 0.033 a
Tuono	In-shell	Blanching	0.465, 0.031 ab	0.448, 0.033 b
Vinci a tutti	In-shell	Blanching	0.488, 0.033 a	0.454, 0.029 b
Genco	In-shell	Roasting	0.834, 0.031a	0.822, 0.028 a
Tuono	In-shell	Roasting	0.814, 0.034 b	0.808, 0.030 b
Vinci a tutti	In-shell	Roasting	0.845, 0.031 a	0.796, 0.031 b
Genco	In-vacuum	Blanching	0.498, 0.035 a	0.466, 0.031 a
Tuono	In-vacuum	Blanching	0.459, 0.031 b	0.449, 0.032 b
Vinci a tutti	In-vacuum	Blanching	0.498, 0.033 a	0.448, 0.028 b
Genco	In-vacuum	Roasting	0.832, 0.031a	0.818, 0.024 a
Tuono	In-vacuum	Roasting	0.821, 0.034a	0.811, 0.031 a
Vinci a tutti	In-vacuum	Roasting	0.855, 0.031 a	0.788, 0.030 b

*dah, days after harvest—Different letters between cultivars within the same storage duration and post-harvest treatment evidence differences statistically significant per P < 0.01 (Tukey's test)*.

Almonds in shell are able to better maintain the phenolic and antioxidant framework, requires less energy but it is much more challenging for the space needed in dedicated environments and with controlled temperature. The storage of almonds in vacuum, however, requires much less space and a prompt response to market needs; however, almonds must be stored under full control of the cold chain, with controlled humidity and temperature, and at the same time it determines a more complex and articulated management of the shell that would be produced all at once, not gradually but just immediately after harvesting, flooding the companies that, on the contrary, would manage the disposal or reuse in a more gradual way according to many industrial hypothesis (50). It remains to be verified, indeed, whether the short- and medium-term storage at low temperature of vacuum shelled almonds may have affected the polyphenolic profile; it appears quite conflicting and, above all, it does not seem to respond to well-defined and certain biological indications (51, 52). In nuts, storage at low temperatures has evidenced some encouraging results in terms of preservation of bioactive substances (53).

## Conclusions

The analysis performed has confirmed the positive relationship between the content of phenolic compounds and antioxidant capacity, so far analyzed in other species such as table olives (54) pitted table olives (55) and hazelnuts (56). As it is known, most of the phenolic compounds contained in almonds are localized in the skin (23) and now it has been shown how their concentration varies in relation to the procedure applied to separate the skin from the seed. The reduction of this potential in relation to industrial treatment depends substantially on the solubilization of these substances in the water used in the peeling process. From here it follows that an accurate analysis of the potential reuse of this water represents a further development of knowledge and added value for the by-products of almond processing in order to ensure the production of bioactive substances for use in the agri-food industry.

Independently of industrial process used for the separation from skin, the preservation in shell has been found to be more effective in order to reduce the loss of bio-active substances over time.

Nowadays, producers try to find solutions which are particularly appreciated by consumers not only in terms of taste attribute but also in relation to the potentiality of food to contribute to a general sustainability also for the environment. The increased awareness that consumption of almonds with skin is often associated with higher nutraceutical quality, as well as taste, is supported by the results of the present research in terms of high phenolics content in the skin and the possibility to preserve them during long-term storage periods. These findings also stimulates further research hypotheses that will be investigated with additional experimental approaches.

## Data Availability Statement

The raw data supporting the conclusions of this article will be made available by the authors, without undue reservation.

## Author Contributions

FS and CP were responsible for the design of the study. CC participated in data collection, laboratory and statistical analyses. All authors contributed to the article and approved the submitted version.

## Conflict of Interest

The authors declare that the research was conducted in the absence of any commercial or financial relationships that could be construed as a potential conflict of interest.
